# The Effect of Social Distance on Intertemporal Choice of Reward Processing: An Event-Related Potentials Study

**DOI:** 10.3389/fnhum.2021.712194

**Published:** 2021-07-21

**Authors:** Shulin Tang, Jie Guo, Bing Li, Zhikai Song

**Affiliations:** ^1^School of Economics and Management, Harbin Engineering University, Harbin, China; ^2^Neural Industrial Engineering Lab SEM, Harbin Engineering University, Harbin, China

**Keywords:** social distance, intertemporal choice, ERPs, P3a, P3b, cognitive control

## Abstract

Social factors can affect the processing of intertemporal choice, but the influence of social distance on the rewarding process of intertemporal choice is unclear. Therefore, by designing a novel cognitive resource competition paradigm for undifferentiated intertemporal choice, this article aims to explore the influence of social distance on intertemporal choice reward processing at the electrophysiological level. It was found that compared with the stranger condition, P3a is greater in the friend condition, which means social distance is evaluated in the early stage. In addition, different brain regions in the early stages are taking charge of processing the soon-but-small (SS) and later-but-lager (LL) reward in intertemporal choice. There is an interaction effect between social distance (friend vs. stranger) and intertemporal choice (SS reward vs. LL reward) on P3b. Under friend conditions, the P3b induced by LL reward is more positive than SS reward. Under the condition of choosing the LL reward, the P3b induced by friend is more positive than stranger. This result shows that in the latter stage of reward processing, the evaluation process of time discounting is less sensitive in LL reward for friend caused by lack of cognitive resources which is occupied when dealing with social distance in advance, and thus the degree of time discount was reduced. These findings demonstrate that P3b is the key index of time discounting and immediate and delayed rewards are valued in different brain regions.

## Introduction

Intertemporal choices refer to the process in which a person chooses by weighing the costs, results, and benefits that occur at different time points (Frederick et al., 2002; Moreira et al., 2016). Although the Exponential discounting model of neoclassical economic theory suggests that the rational choice for an individual is a Larger-but-Later reward (LL; Samuelson, 1937), a large body of research shows that people have an overwhelming preference for Smaller-but-Soon rewards (SS; O’Donoghue and Rabin, 1999; Cherniawsky and Holroyd, 2013). Therefore, behavioral economists proposed the hyperbolic discounting model (Mazur, 1987) and the quasi-hyperbolic discounting model (Laibson, 1997) to predict decision-makers’ choices. The discount rate K is used to measure the degree of personal temporal discount. The higher the value of K, the greater the discount for delayed rewards, that is, they prefer SS rewards more. Intertemporal choice is not only related to individual interests but also related to important decisions of the national economy and people’s livelihood. For example, does an individual choose to indulge in high-calorie foods or choose healthier foods to keep fit? Do the factory decision-makers choose random discharge to save costs or centralize treatment to protect the environment?

Many factors affected intertemporal choice, not only the differences between individuals (Elton et al., 2017; Xia et al., 2017; Liu et al., 2019) but also the social environment in which they live (Li et al., 2012; Calluso et al., 2017; Yi et al., 2020; Yu et al., 2020; De Oliveira and Jacobson, 2021). Among those, social distance is a very important factor. It describes the emotional intimacy between self and others, inside and outside the group, and is the physical manifestation of psychological distance (Yaacov and Nira, 2010). For individuals, friends are at a low level of social distance, while strangers are at a high level of social distance. De Oliveira and Jacobson (2021) explored whether individuals have made intertemporal choices for people of different social distances by allocating intertemporal working hours. Results found that individuals make sub-optimal decisions that are more impatient for strangers. But in the case of lower social distance, the bias of this sub-optimal choice will be alleviated, and the decision-making will be more rational. However, Ma et al. (2011) studied how interpersonal familiarity and self-participation regulate the time course of the neural response to the gains and losses of friends or strangers, and found that there is a significant difference in the neural response of the subjects observing the gains and losses of friends and strangers, But this difference is only significant when the participant does not participate in the gambling game. In addition, there are not only situations in which one makes the intertemporal choice in life, but also situations in which others make the intertemporal choice for you. Therefore, we need to design a novel experimental paradigm that allows subjects to watch without making decisions to explore the influence of social distance on intertemporal choice.

Recent fMRI studies of intertemporal choice of neural associations have generated two different explanations. McClure et al. (2004) suggest that there are two separate neural systems for evaluating SS and LL rewards. System 1 is parts of the midbrain limbic reward system innervated by rich dopaminergic nerves, and the activation of this system is related to SS reward. It is a low-level automatic processing system, including the ventral striatum, medial orbital frontal cortex, medial prefrontal cortex, posterior cingulate cortex, and left posterior hippocampus. System 2 is composed of the lateral prefrontal and parietal cortex, which involves higher levels of deliberative processes and cognitive control, and this system is active in all intertemporal choices (McClure et al., 2004, 2007). In contrast, an alternative interpretation supports a single system for time processing. Kable and Glimcher (2007) showed that the neural activity of the ventral striatum, medial prefrontal cortex, and posterior cingulate gyrus tracked the subjective value of LL reward. Hence, these brain regions encode the subjective value of SS and LL rewards on a common scale, disproving the opinion that these brain regions only evaluated SS reward. In addition, Monterosso and Luo (2010) put forward an argument against the competition of dual-system evaluation systems, pointing out that the processing of SS reward mainly relies on complex cognitive systems, but these structures influence intertemporal choices by regulating the midbrain limbic reward system rather than competing with it to control reward and motivation. Therefore, there is still a lot of controversy regarding the neural processing behind intertemporal choices.

In addition, a large number of studies have taken advantage of the high temporal resolution of event-related potentials to explore the temporal process in intertemporal choice (Li et al., 2012; Cherniawsky and Holroyd, 2013; Gui et al., 2016; Liu et al., 2019). For example, Li et al. (2012) used ERPs technology to explore the impact of earthquakes on personal time discounting, indicating that post-disaster decision-making may not be rational in the dual-process model of decision-making, involving more emotional thinking (System 1) and less rational thinking (System 2). Liu et al. (2019) explored the behavioral and neural correlates of food-related decisions in overweight and normal-weight adults, and found that overweight adults are more impulsive than normal adults, may allocate more cognitive resources to food-related stimuli, and involve higher levels of cognitive processes. Qu et al. (2013) explored the impact of delay on outcome evaluation and found that time discounting in the early stages of outcome evaluation can be coded in the components of ERPs. Patalano et al. (2018) also explored the effect of gratitude intervention on the modulation of P3 amplitude in the temporal discounting task.

Considering the advantages of event-related potentials with high time resolution, this study uses ERPs technology to explore the influence of social distance on intertemporal choice reward processing. The current research explores the influence of social distance on intertemporal choice by watching different decision-makers (friends or strangers) make decisions about their own indifferent intertemporal choice. From the previous ERPs research on intertemporal choice reward evaluation, it can be known that P300 is the most concerning component (Li et al., 2012; Gui et al., 2016; Patalano et al., 2018). The P300 component scalp is usually distributed on the midline electrodes (Fz, Cz, Pz), and the amplitude of the electrodes from the frontal lobe to the parietal lobe increases gradually (Johnson, 1993). According to the time course, P300 can be divided into P3a and P3b (Squires et al., 1975). P3a is a component with a short latency, which is peaking at around 200–280 ms after stimulus onset. The scalp is widely distributed, with the largest amplitude located in the back of the frontal lobe. P3a is related to frontal lobe attention and working memory and is regulated by dopamine activity, reflecting the bottom-up attention processing mechanism of the forebrain area driven by stimuli (Friedman et al., 2001; Polich, 2007). P3b has a longer latency than P3a, and it is usually distributed in the central-parietal region peaking at 300–600 ms after the onset of stimuli. P3b reflects the task-driven top-down attention and memory mechanism of the temporal-parietal area, which is regulated by the norepinephrine system (Friedman et al., 2001; Sander et al., 2005; Polich, 2007). Moreover, it has been found that it is very sensitive to the amount of the monetary reward in the card gambling task (Nick and Sanfey, 2004) and is related to the motivational relevance of the stimulus (Sander et al., 2005). P3a and P3b originate from the activation of the frontal and temporal-parietal lobes and form a pathway in the frontal and temporal-parietal regions (Polich, 2007).

Therefore, the current research intends to explore the temporal dynamics of the influence of social distance on intertemporal choice. Considering that the intertemporal choice set in this experiment is not different for the subjects, it is assumed that in the early stage of reward processing, only the social information of social distance is processed. In the detailed processing stage of rewards, social information will be integrated with time information, thereby affecting the individual’s preference for intertemporal choice. Since P3a is related to top-down attention processing, we hypothesize that P3a can reflect the early processing of social distance, that is, there will be differences in neural responses when watching friends and strangers make intertemporal choices. P3b is related to bottom-up attention and memory processing. Previous studies have shown that it is thought to reflect the attention assigned to the outcome, as well as the motivational/affective salience of the outcome, and a greater positive value of P3 amplitude indicates that more attention resources are used for stimulation and enhance the activation of the motivation system (Broyd et al., 2012; Wang et al., 2018). We hypothesize that in the detailed processing stage of rewards, P3b reflects the fine evaluation of social distance and intertemporal choice. If social distance affects the processing of intertemporal choice, it will be reflected through the interaction of social distance and time delay. If there is no effect, there will be no significant difference between the ERP waveforms at this stage.

## Materials and Methods

### Participants

Eleven pairs of subjects (six male pairs) were recruited from undergraduate and graduate students of Harbin Engineering University, ranging in age from 18 to 28 years old (*M* = 22.2, *SD* = ± 2.30). Due to excessive artifacts during EEG recording, two participants’ (two females) data were discarded, and 20 subjects^1^ (12 males and eight females) were included in the final analysis. Each pair of participants are self-reported good friends, gender-matched, and know each other. In addition, two participants (one male and one female) were recruited as strangers, who did not know each other, and were gender-matched with each pair of participants. All of them reported normal vision or correction and no mental or neurological illness. All participants signed an informed consent form before the study and could withdraw from the study at any time to protect their rights. This research scheme was approved by the local ethics committee and is in line with the latest version of the Helsinki Declaration. Participants were paid 30 yuan (about $4.50) at the end of the experiment.

### Experimental Stimuli

**Figure 1:**
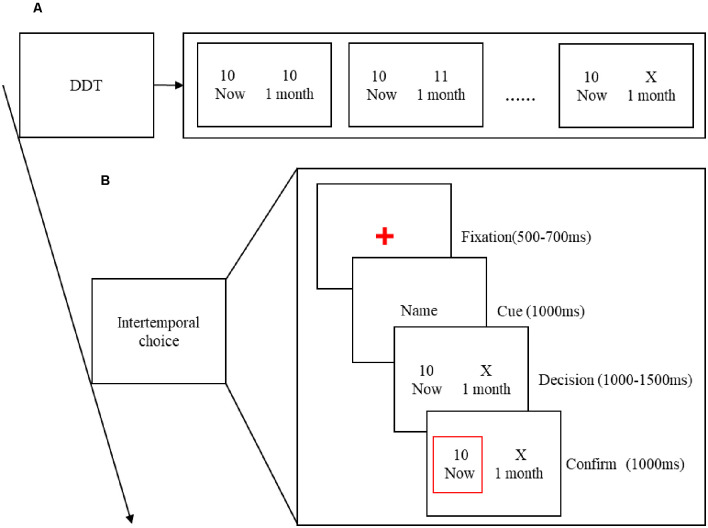
**(A)** The process of the Delayed Discount Task (DDT). **(B)** Time course of a single trial. Each trial began with a red fixation, randomized between 500 and 700 ms. Followed by the name of decision-maker for 1,000 ms. Then, the participant’s indifferent intertemporal choice presented at a random time of 1,000–1,500 ms. Finally, the decision-maker’s choice of 1,000 ms is shown in the red border.

An adapted version of the Delayed Discount Task (DDT) was used to measure the participants’ indifference points before the EEG recordings were performed (Myerson and Green, 1995), thereby eliminating the influence of the two factors (delay time and reward amount) of intertemporal choice. Among them, the SS reward is always set to get 10 yuan immediately, and the delay time of the LL reward is always 1 month. Because different individuals have different degrees of time discounting, in order to eliminate the influence of individual differences on this experiment, the amount of LL reward is set according to the participant’s personal time perception value. The specific setting method is as follows. There are two types of cards on the table in front of the participants. One is an immediate fixed amount of reward (that is, you get 10 yuan immediately), and the other is a variable amount that you get with a delay of 1 month (gradually increases from 10 yuan). The experimenter changes the card until the subject’s preference is shifted. When the subject’s preference is deflected, the amount of X yuan is used as the subject’s LL reward. In other words, for this participant, getting 10 yuan immediately is equivalent to getting X yuan after 1 month later. Therefore, this intertemporal choice is regarded as the indifference point of the subject. As shown in Figure 1A.

### Procedure

Due to the limited EEG collection equipment in the laboratory, when a pair of gender-matched friends come to the laboratory, they decide who will start the experiment first. His/her friend and a stranger who gender-matched go to the next room to act as decision-makers to improve the credibility of the participants. Participants carried out the experiment in a closed and quiet environment, sitting in the most comfortable position about 1 m away from the monitor and wearing an electrode cap. Instructions will be given to the participants to introduce the experiment process before the experiment begins. Before the formal experiment, participants were given 10 practice experiments to familiarize themselves with the operation process of the experiment. In the formal experiment, a fixed cross was first presented at a random time of 500–700 ms. Then, the name of the decision-maker who made the selection in this experiment was presented for 1,000 ms to prompt friends or strangers for intertemporal choices task. Then, the participants’ indifferences intertemporal choices option is presented at a random time of 1,000–1,500 ms, and the decision-maker’s choice of 1,000 ms is shown in the red border (Figure 1B). The formal experiment consisted of three blocks, each with 40 trials, for a total of 120 trials. In fact, the computer program predetermines the number of trials set under each condition, which are 30. And presented them in a pseudo-random order that ensures that two adjacent trials are not the same.

### EEG Recording and Analysis

EEG was recorded continuously (bandpass filter 0.05–100 HZ, sampling rate 1,000 HZ) with the NeuroScan Synamp2 Amplifier (Scan 4.3.1, Neurosoft Labs Inc., USA). The electrode caps with 64 Ag/Agcl electrodes and the other two electrode caps placed on the mastoid (behind the ear) are installed following the international standard 10–20 system. Use the tip of the nose as an online reference. All channels were offline re-referenced to the average of the left and right mastoid references. The electrode between FPz and Fz was applied as ground. The vertical EOG and horizontal EOG were recorded by two electrodes located above and below the left eye 10 mm and lateral electrodes on the outer canthi of both eyes. All electrode impedances were maintained below 10 KΩ.

EEG data were analyzed offline using NeuroScan version 4.3 software. During the analysis, the EEG data were averaged by different conditions. The ERPs were filtered with a 30 HZ (24 dB/octave) low-pass filter. For ERP analyses, the data were segmented for the epoch from 200 ms before the onset of the stimulus to 800 ms after its onset, and the whole epoch was baseline-corrected by the 200 ms pre-stimulus onset. Ocular artifacts were corrected with an eye-movement correction algorithm proposed by Gratton et al. (1983). Any epoch containing amplifier clippings, bursts of electromyography activity, or maximum EEG Voltage exceeding a threshold of ±100 μV were excluded from further analysis. The acceptance rate of four conditions are 96.7% (Friend_SS), 98.1% (Friend_LL), 98.5% (Stranger_SS), and 98.1% (Stranger_LL), respectively. The number of trials under each condition is more than 25, which can be further analyzed (Patalano et al., 2018). The time window of 200–300 and 300–400 ms after the stimulus onset were chosen for the analysis of P3a and P3b based on previous studies and visual observation, respectively. The nine electrodes (C1, CZ, C2, CP1, CPZ, CP2, P1, PZ, P2) in the central-parietal area were used to analyze P3a and P3b. A 2 (social distance: friend vs. stranger) × 2 (intertemporal choice: SS reward vs. LL reward) × 9 (electrodes) three-way repeated-measures analysis of variance was used.

## Results

### P3a

**Figure 2:**
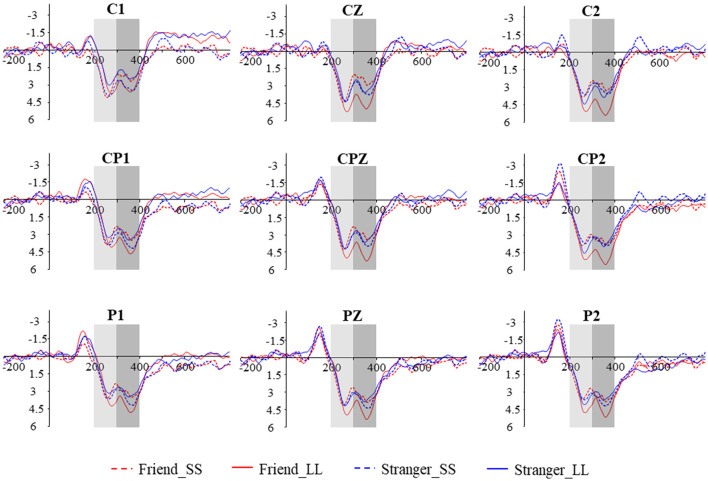
Grand-averaged ERP waveforms from nine electrodes in four conditions. The time window of P3a and P3b are 200–300 ms and 300–400 ms, respectively.

We performed a 2 (social distance: friend vs. stranger) × 2 (intertemporal choice: SS reward vs. LL reward) × 9 (electrodes: C1, CZ, C2, CP1, CPZ, CP2, P1, PZ, P2) repeated measures analysis of variance (ANOVA) for P3a, the results show that the main effect of the agency is significant (*F*_(1,19)_ = 7.081, *p* = 0.015, $\eta_{p}^{2}$ = 0.272), The P3a induced by friend condition (5.551 ± 0.411 μV) was greater than that induced by stranger condition (5.004 ± 0.426 μV; Figures 2, 5A). In addition, the main effect of the electrode is also significant (*F*_(8,152)_ = 2.441, *p* = 0.016, $\eta_{p}^{2}$ = 0.114). But the main effect of the option (*F*_(1,19)_ = 1.228, *p* = 0.282, $\eta_{p}^{2}$ = 0.061) and the interaction between agency and option (*F*_(1,19)_ = 0.462, *p* = 0.505, $\eta_{p}^{2}$ = 0.024) have not been found. In addition, the results also show that the interaction between option and electrodes is significant (*F*_(3.283,62.370)_ = 6.982, *p* < 0.001, $\eta_{p}^{2}$ = 0.269). Other interactions with the electrode are not significant (*p*s > 0.05).

**Figure 3:**
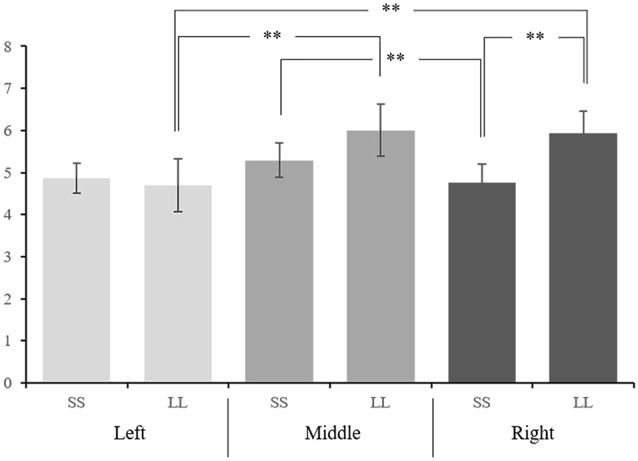
The interaction effect between option and hemisphere of mean amplitudes of P3a at nine electrodes. **p < 0.05.

**Figure 4:**
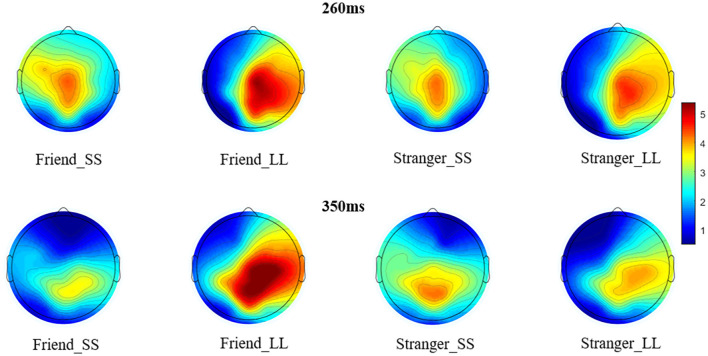
The brain topographic voltage maps at 260 ms and 350 ms under four conditions.

**Figure 5:**
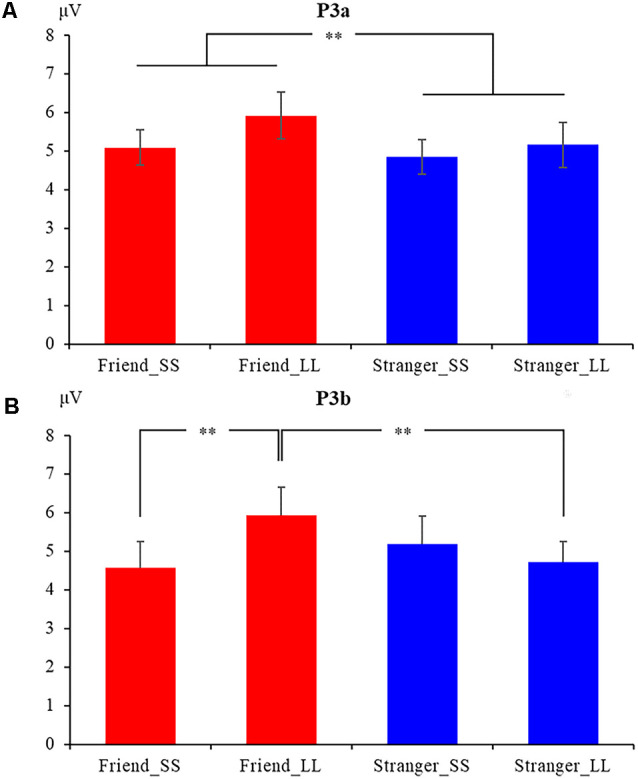
The mean amplitudes of P3a **(A)** and P3b **(B)** at nine electrodes under four conditions. **p < 0.05.

In order to further explore the interaction between option and electrodes, we divided the electrodes into left hemisphere (C1, CP1, P1), middle hemisphere (CZ, CPZ, PZ), and right hemisphere (C2, CP2, P2) for hemisphere effect analysis. The interaction between option and hemisphere is significant (*F*_(1.194,22.685)_ = 13.995, *p* = 0.001, $\eta_{p}^{2}$ = 0.424; Figure 3). Further simple effect analysis showed that on the right hemisphere, the P3a amplitude induced by LL reward (5.936 ± 0.524 μV) was significantly greater than SS reward (4.757 ± 0.445 μV, *p* = 0.018), while on the left and middle hemisphere we didn’t find any significant difference (*p*_left_ = 0.778, *p*_middle_ = 0.198). In addition, under the SS reward condition, the P3a amplitude on middle hemisphere (5.287 ± 0.410 μV) is significantly greater than right hemisphere (4.757 ± 0.445 μV, *p* = 0.019). Under LL reward condition, the P3a amplitude on the middle and right hemisphere (middle: 6.005 ± 0.620 μV; right: 5.936 ± 0.524 μV) are significantly larger than on the left hemisphere (4.691 ± 0.628 μV, *p*_left-middle_ < 0.001, *p*_left-right_ = 0.002), while on the middle and right hemisphere we didn’t find significant difference (*p* > 0.05). According to the latency of P3a, the brain topography under four conditions at 260 ms was drawn (Figure 4).

### P3b

We also performed 2 (social distance: friend vs. stranger) × 2 (intertemporal choice: SS reward vs. LL reward) × 9 (electrodes: C1, CZ, C2, CP1, CPZ, CP2, P1, PZ, P2) repeated measures ANOVA for P3b. The results show that the main effect of the electrode is significant (*F*_(8,152)_ = 2.630, *p* = 0.010, $\eta_{p}^{2}$ = 0.122), while the agency (*F*_(1,19)_ = 1.138, *p* = 0.299, $\eta_{p}^{2}$ = 0.057) and option (*F*_(1,19)_ = 0.897, *p* = 0.360, $\eta_{p}^{2}$ = 0.044) are not significant. More importantly, the results found that the interaction between agency and option is significant (*F*_(1,19)_ = 5.881, *p* = 0.025, $\eta_{p}^{2}$ = 0.236). Further simple effect analysis showed that the P3b produced by LL reward (5.944 ± 0.728 μV) was significantly greater than the SS reward (4.584 ± 0.673 μV, *p* = 0.037) under friend conditions, while under stranger conditions, the difference was not found (*p* = 0.465). What’s more, under LL reward conditions, the P3bproduced by friend (5.944 ± 0.728 μV) was significantly greater than stranger (4.729 ± 0.534 μV, *p* = 0.012), while no significant difference was found between friend and stranger under the condition of SS reward (*p* = 0.250), as shown in Figures 2, 5B. The interaction between agency × electrode and option × electrode are not significant. According to the latency of P3b, the brain topography of the four conditions at 350 ms was drawn (Figure 4).

## Discussion

This study explores the effect of social distance on intertemporal choice. Electrophysiological results found that the main effect of the P3a agency is significant. The amplitude of P3a generated under the condition of watching friends’ decision-making is greater than that of watching strangers’ decision-making, indicating that the social distance effect is activated when the subjects watch intertemporal choice. Interestingly, we also found the interaction between option and hemisphere. On the right hemisphere, the P3a caused by LL reward is greater than the SS reward. Under the SS reward condition, the P3a amplitude at the middle hemisphere is significantly greater than the right hemisphere. What’s more, under the LL reward condition, the P3a amplitude at the middle and right hemispheres is greater than the left hemisphere. In the phase of detailed evaluation of outcome, social information and temporal information are integrated and reflected through the interaction of P3b. Under friend conditions, it was observed that the amplitude of P3b generated when the decision-maker chose the LL reward was greater than the SS reward, but this effect was not observed under stranger conditions. When decision-maker choosing LL reward, the P3b amplitude elicited by observing friend’s decision is greater than observing stranger’s choice, but this effect was not observed in SS reward.

P3a reflects the bottom-up attention processing mechanism driven by stimuli (Friedman et al., 2001; Polich, 2007). The amplitude of P3a generated under friend conditions is greater than stranger conditions, indicating that the bottom-up attention processing system is more activated under friend conditions and more attention resources are devoted to friends. In other words, they are more concerned about the choices made by friends than strangers, which is consistent with previous research (Ma et al., 2011; Cui et al., 2017; Jin et al., 2020). The early stage of the evaluation of rewards is manipulated not only by the amount of reward but also by emotion. Our results showed that there is a main effect on social distance in the early stage, so we infer that it includes an unconscious attention processing such as emotional processing merged with reward valence processing. Since the intertemporal choices in this experiment are set as the indifference option, it will not have a main effect on the intertemporal choice, which also verifies the successful manipulation of the indifference point in the experiment. The interaction between social distance and intertemporal choice requires a more detailed processing system and fine-grained analysis, which will be reflected through P3b in the later stage of the reward outcome evaluation. Interestingly, we found the interaction between option and hemisphere on the P3a component. Previous fMRI studies have shown that the occipital and parietal cortex are activated for the delayed option (McClure et al., 2004; Ballard and Knutson, 2009; Faralla et al., 2015). The research of Ballard and Knutson (2009) showed that the increase in delayed reward amplitude is positively correlated with the activation of right Nucleus accumbens (NAcc), medial prefrontal cortex (MPFC), and posterior cingulate gyrus, while the increase in delayed reward is negatively correlated with the activation of the left dorsolateral prefrontal cortex (DLPFC), right posterior parietal cortex (PPC), and left temporal-parietal junction (TPJ). In this study, the processing of intertemporal choice activated different brain regions, thus providing evidence for the dual-system processing theory (McClure et al., 2004, 2007). The LL reward elicits a more positive P3a amplitude on the right hemisphere, indicating that the right parietal lobe is more activated in the LL reward condition and more cognitive resources are used to dealing with a better offer. But overall, the main effect of the option was not found in the P3a component. It is very likely that at the early evaluating stage, only certain risk information i.e., reward amount, social distance is encoded and processed, and the processing of uncertain risk information such as time discounting is hanged up and will be initiated after certain risk information had been processed. So we also speculate that different brain regions have initiated the processing of immediate and delayed reward in intertemporal choice respectively, the left DLPFC is taking charge of evaluating immediate reward and the right temporal occipital lobe is responding for a delayed reward. But further research is needed using fMRI technology with high spatial resolution.

Previous studies have found that the main source of P3a is the frontal lobe (Polich, 2007; Ernst and Steinhauser, 2020), while this study found that the source of P3a is the central parietal region. This difference may be due to the different experimental paradigms. Previous studies have adopted the oddball paradigm, and non-target stimuli that occur infrequently can cause P3a (Polich, 2007; Nieuwenhuis et al., 2011; Wei et al., 2020). However, this study did not adopt the oddball paradigm, instead of the S1–S2 paradigm. Because the subjects have different social relationships with decision-makers (S1 stimulus), which affects the processing of intertemporal choices (S2 stimulus) process. P3a just reflects the subjects’ perception and processing of different decision-makers (S1 stimuli) and didn’t find any significant effects on intertemporal decision-making.

P3b reflects task-driven top-down mechanisms of attention and memory in the temporoparietal region (Polich, 2007). The effect of P3b is consistent with that of P300 or P3 in most studies (Riepl et al., 2016; Kiata et al., 2018). Many previous studies have shown that P3b amplitude is sensitive to reward valence and amount, which is related to the redistribution of attention resources and the motivational significance of stimuli (Linden, 2005; Sander et al., 2005; Polich, 2007; Yuanyuan et al., 2017). In the present study, it was found that under friend conditions, the indifferent intertemporal choices process produced different P3b amplitudes. Compared with the SS option, the LL option produced larger P3b amplitudes, while no such effect was found under the stranger conditions. This result shows that under friend conditions, the indifference intertemporal choice before the experiment was re-evaluated, and compared with the SS reward, more attention resources were allocated to the LL reward. It indicates that the LL option is more motivational in the friend condition. In addition, under the condition of LL reward, compared with stranger, the decision of a friend caused a larger P3b amplitude, but this effect was not found under the condition of SS reward. This result shows that while more attention resources are devoted to the LL reward, it does not reduce the attention resources devoted to the SS reward. Together, it can be shown that in the detailed stage of the reward evaluation, compared with stranger condition, the LL reward under friend condition has distributed more attention resources and stronger motivation. Compared with strangers, friends are close psychological distances for the subjects. According to the construct level theory, objects at a close psychological distance tend to be inclined to a specific and complex representation of a low level of construct (Yaacov and Nira, 2010). Therefore, the friend condition in the social distancing stage occupies a lot of cognitive resources, which leads to insufficient cognitive resources to process time discount information in the detailed outcome stage, thus reducing the degree of time discounting. Moreover, when processing the SS, there is no need to discount the time, so the difference is only in the stage of the LL reward processing.

In addition, the hyperbolic discounting function shows that with the extension of LL reward delivery time, the degree of discount of LL rewards by individuals also increases, and they prefer to get rewards immediately (Laibson, 1997) so that the attention resources are less devoted to LL rewards. The results of this study support the explanation, compared with a stranger, less cognitive resources are assigned to time discounting under friend condition because subjects need extra cognitive resources to evaluate the friendship, thus time discounting will be less sensitive for observing friend’s delayed reward. The reason is that the shortage of cognitive resources will lead to irrational time perception that subjects will underestimate time span of delayed reward option. The shorter time period causes a smaller time discounting rate K because people would think they will get the reward earlier than 1 month set in the task, so the utility will increase for LL reward in friend condition which is reflected in the result that the P3b of LL reward in friend condition is more positive than other three conditions.

Previous studies have also shown that individuals with poor self-control are more likely to choose the immediate reward option (Jimura et al., 2013; Liu et al., 2019), that is, more impulsive people have a higher discount rate to time. The key phenomenon of impulse control is preference reversal (Ashe and Wilson, 2020). The intertemporal choices in this study are indistinguishable for the subjects, but the amplitude of P3b under the conditions of friends and strangers has a significant difference. However, this change was not found in stranger condition. It shows that under the condition of friends, in the early stage of the result evaluation, it is necessary to process the social information of close psychological distance, which occupies a certain cognitive resource. Therefore, in the detailed stage of the result evaluation, there are not enough cognitive resources to process the time discount. Finally, it is insensitive to time discounting, which reduces the degree of time discounting. Therefore, individuals deflected the intertemporal option with no difference, that is, under the friend condition, subjects preferred the LL reward, which was also reflected by the more positive P3b amplitude under this condition. Therefore, the results of this study suggest that friends can reduce individuals’ discounting of time and enhance self-control, thus having more rational cognitive processing.

According to the theory of dual-system processing, there are two different assumptions in this study. The first hypothesis is that the empathy effect of social distance as an emotion activates the emotional system in the dual-system, that is, the limbic upper limb system rich in midbrain dopaminergic innervation. These systems are related to impulsivity (McClure et al., 2004, 2007). Therefore, in the experiment, it should be observed that the subjects’ preference for SS reward increases so that more attention resources are devoted to SS reward, but the results we observe are contrary to this hypothesis. The second hypothesis is that the social factor of social distance regulates the subjects’ handling of intertemporal choices. Under the condition of friends, due to the empathy effect, the individual needs part of cognitive resources to process the specific and complex near psychological distance, thus the individual’s perception of time information in the detailed stage of reward processing is reduced, and the degree of time discount is reduced. When the friend decision-maker selects the LL option, it launched the dual-system in the higher levels of deliberative processes and cognitive control system, the system with a high level of reasoning and the ability of future plans, so participants should be observed in the experiment of increased preference for LL reward, which devoted more attention resources to the LL reward. Our experimental results are consistent with the second hypothesis, suggesting that the effect of social distance activates System 2 of the dual-systems, thereby reducing individuals’ discounting of time and improving their ability to control cognition. Of course, to be cautious in making such an explanation, further fMRI studies are needed to process the brain regions of SS and LL reward under different social distance conditions.

However, we acknowledge that this study has some limitations. In the intertemporal choices, the amount of the SS reward was set to 10 yuan, and the time delay of the LL reward was set to 1 month. This was because setting only one intertemporal choice was convenient for the subjects to remember their indifferent point accurately, and it would not occupy too many memory resources. Indeed, further research is needed to determine whether the results we found in one intertemporal choice pair have the same effect in other intertemporal choices, and a series of indifference points can be set in future studies to test the stability of the results of this study. In addition, previous studies have found that the brain’s response to gain and loss in intertemporal choices is asymmetric (Qu et al., 2013; Lei et al., 2018). People usually discount losses at a lower rate than gains, which is the sign effect (Loewenstein, 1987). However, in this study, only gains were discussed. Therefore, in future studies, we can explore whether the effects of gains and losses of social distance also have different effects in intertemporal choice.

To conclude, we designed a novel undifferentiated intertemporal choice experiment paradigm to investigate the effect of social distance on intertemporal choice at the electrophysiological level. In the early stage of outcome processing, the social distance was processed, and the P3a component was reflected in friends compared to strangers, which is a top-down attention processing. In addition, different brain regions in the early stages process the soon-but-small (SS) and later-but-lager (LL) reward in intertemporal choice. In the detailed phase of reward processing, social distance affects the processing of intertemporal choice, which is reflected by the interaction between social distance and intertemporal choice on P3b. Under the condition of friends choose LL reward has caused more positive P3b amplitude. This result shows that in the latter stage of reward processing, the evaluation process of time discounting is less sensitive in LL condition for friend caused by lack of cognitive resources which is occupied when dealing with social distance in advance. These findings demonstrate that P3b is the key index of time discounting and immediate and delayed rewards are valued in different brain regions.

## Data Availability Statement

The raw data supporting the conclusions of this article will be made available by the authors, without undue reservation.

## Ethics Statement

The studies involving human participants were reviewed and approved by Ethics Committee of the School of Economics and Management, Harbin Engineering University. The patients/participants provided their written informed consent to participate in this study.

## Author Contributions

ST, JG, and BL conceived and designed the research. JG and ZS performed the experiments. JG analyzed the data and wrote the manuscript. ST and BL reviewed the manuscript. All authors contributed to the article and approved the submitted version.

## Conflict of Interest

The authors declare that the research was conducted in the absence of any commercial or financial relationships that could be construed as a potential conflict of interest.

## Footnotes

^1^Simple sizes in 13 to 28 ranges are suitable for ERP studies involving multiple trials and posterior analyses (Jing et al., 2019).
